# Supplementary material for: scaDA: A novel statistical method for differential analysis of single-cell chromatin accessibility sequencing data
Source: PLoS Comput Biol. 2024 Aug 2;20(8):e1011854. doi: 10.1371/journal.pcbi.1011854 (PMC11324137; doi:10.1371/journal.pcbi.1011854)
Supplement: S1 Text — (PDF) [file pcbi.1011854.s001.pdf]

S1 Text. EM algorithm for ZINB model

The details for estimating mean, prevalence and dispersion parameters of ZINB model using EM algorithm are illustrated as follows.

The complete likelihood function is

$$\begin{aligned} L(\mathbf{y_g}, \mathbf{r_g} \mid \Theta) &= \prod_{i=1}^{n} f(y_{gi}, r_{gi}|\Theta) \\ &= \prod_{i=1}^{n} f(y_{gi}|r_{gi}, \Theta) f(r_{gi}|\Theta) \\ &= \prod_{i=1}^{n} f_{NB}(y_{gi}|\mu_g, \phi_g)^{(1-r_{gi})} p_g^{r_{gi}} (1-p_g)^{(1-r_{gi})} \end{aligned} \tag{1}$$

The complete log-likelihood function is

$$\ell_c(\mathbf{y_g}, \mathbf{r_g} \mid \Theta) = \sum_{i=1}^{n} r_{gi} \log(p_g) + (1-r_{gi}) \log(1-p_g) + (1-r_{gi}) \log(f_{NB}(y_{gi}|\mu_g, \phi_g)) \tag{2}$$

The E-step and M-step of the EM algorithm are
E-step:

$$\mathbb{E}[r_{gi}] = \frac{p_g \cdot \mathbf{1}_{(y_{gi}=0)}}{p_g \cdot \mathbf{1}_{(y_{gi}=0)} + (1-p_g) \cdot f_{NB}(0|\mu_g, \phi_g)} \tag{3}$$

M-step:

$$p_g = \frac{1}{n} \sum_{i=1}^{n} \mathbb{E}[r_{gi}] \tag{4}$$

$$\mu_g, \phi_g = \arg\max \sum_{i=1}^{n} (1 - \mathbb{E}[r_{gi}]) \log(f_{NB}(y_{gi}|\mu_g, \phi_g)) \tag{5}$$
