# Supplementary figures and images for: scaDA: A novel statistical method for differential analysis of single-cell chromatin accessibility sequencing data
Source: PLoS Comput Biol. 2024 Aug 2;20(8):e1011854. doi: 10.1371/journal.pcbi.1011854 (PMC11324137; doi:10.1371/journal.pcbi.1011854)

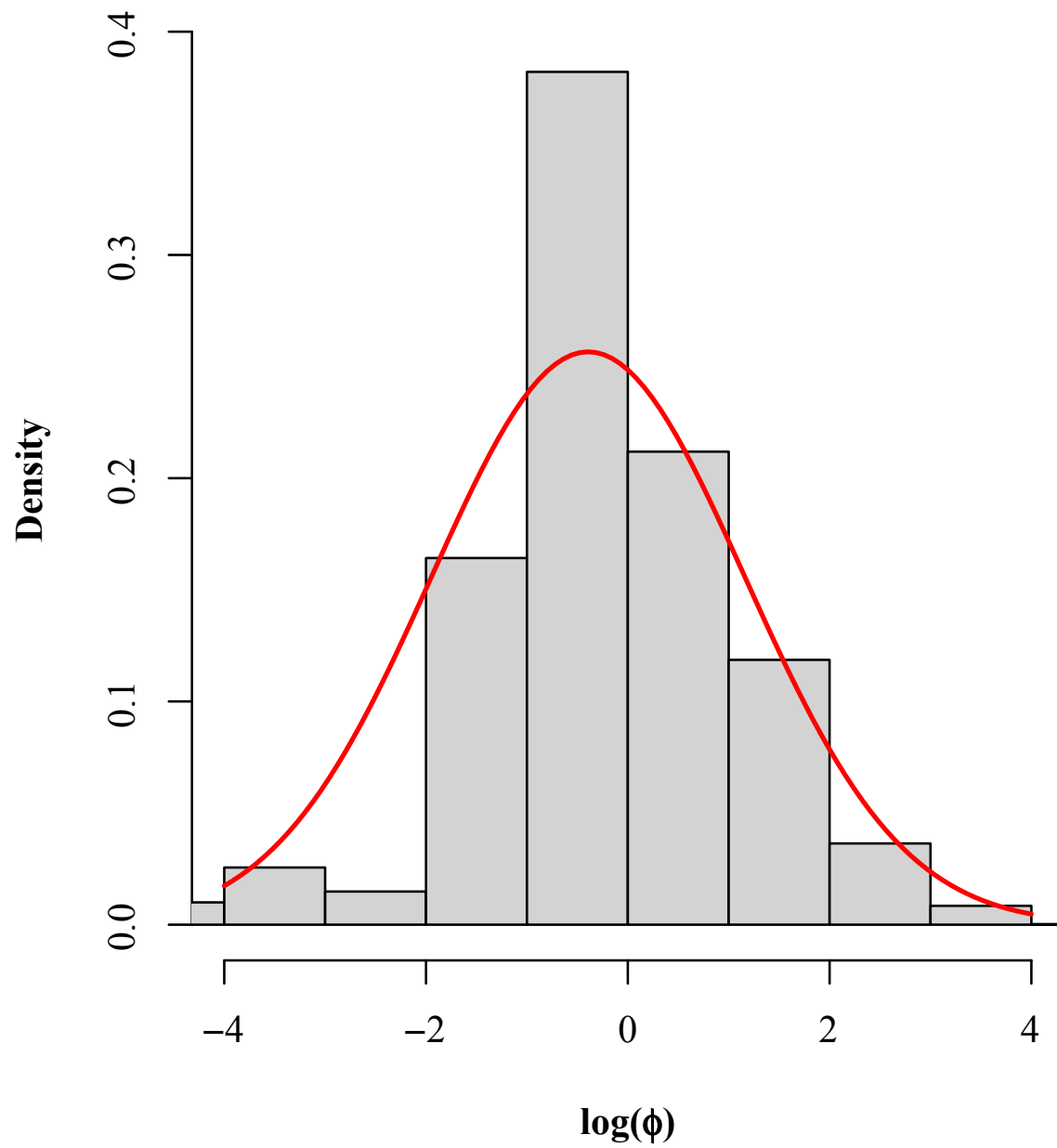

S1 Fig. Histogram of log-transformed dispersion estimates in granule neuron from “Human Brain 3K”

---

Supplement: S1 Fig — (PDF) [file pcbi.1011854.s002.pdf]
