# Supplementary material for: scaDA: A novel statistical method for differential analysis of single-cell chromatin accessibility sequencing data
Source: PLoS Comput Biol. 2024 Aug 2;20(8):e1011854. doi: 10.1371/journal.pcbi.1011854 (PMC11324137; doi:10.1371/journal.pcbi.1011854)
Supplement: S5 Fig — Baseline values of three parameters are estimated in the cell type “Granule neuron” from “Human Brain 3K”. For DA peaks, we fix the parameter values in Group 1 and vary the parameter values in Group 2 with different effect sizes of selected parameters to create power curves. To create parameter values in Group 2, we multiply and divide an effect size in terms of log2 fold change from the baseline values for DA peaks in equal proportion. The log2 fold change of selected parameters is changed from 0.5 to 3.0 with a step size of 0.5. Using the parameter setting in the four scenarios, we assume 20% DA peaks and simulate the read counts based on ZINB for 4000 peaks across 300 cells in each group. A. Scenario 1: only mean difference between two groups B. Scenario 2: only prevalence difference between two groups. C. Scenario 3: only dispersion difference between two groups. D. Scenario 4: difference of all three parameters between two groups. (PDF) [file pcbi.1011854.s006.pdf]

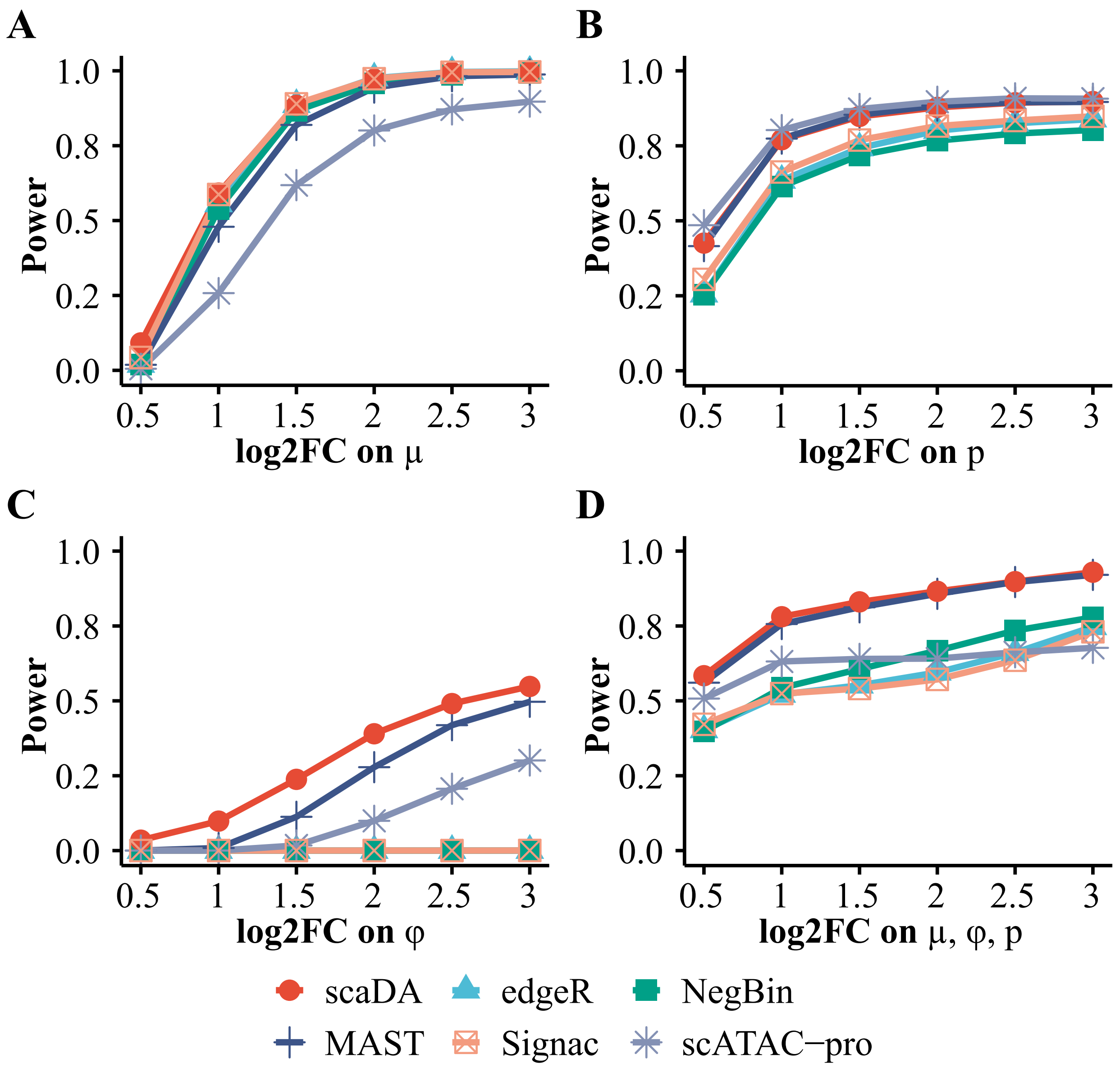

S5 Fig. Power analysis for scaDA and published methods (Sample size 300 in each group). Baseline values of three parameters are estimated in the cell type "Granule neuron" from "Human Brain 3K". For DA peaks, we fix the parameter values in Group 1 and vary the parameter values in Group 2 with different effect sizes of selected parameters to create power curves. To create parameter values in Group 2, we multiply and divide an effect size in terms of log2 fold change from the baseline values for DA peaks in equal proportion. The log2 fold change of selected parameters is changed from 0.5 to 3.0 with a step size of 0.5. Using the parameter setting in the four scenarios, we assume 20% DA peaks and simulate the read counts based on ZINB for 4000 peaks across 300 cells in each group. **A.** Scenario 1: only mean difference between two groups **B.** Scenario 2: only prevalence difference between two groups. **C.** Scenario 3: only dispersion difference between two groups. **D.** Scenario 4: difference of all three parameters between two groups.
