# Supplementary material for: scaDA: A novel statistical method for differential analysis of single-cell chromatin accessibility sequencing data
Source: PLoS Comput Biol. 2024 Aug 2;20(8):e1011854. doi: 10.1371/journal.pcbi.1011854 (PMC11324137; doi:10.1371/journal.pcbi.1011854)
Supplement: S6 Fig — scaDA is compared to both ZINB-based LRTs and published methods. Using the same simulation strategy in Scenario 4 (log2FC = 2.5), we assume 20% DA peaks and simulate the read counts based on ZINB for 4000 peaks across 200 cells in each group. The observed FDR is plotted against the nominal FDR level. A. scaDA is compared to ZINB-based LRT tests. B. scaDA is compared to published methods. (PDF) [file pcbi.1011854.s007.pdf]

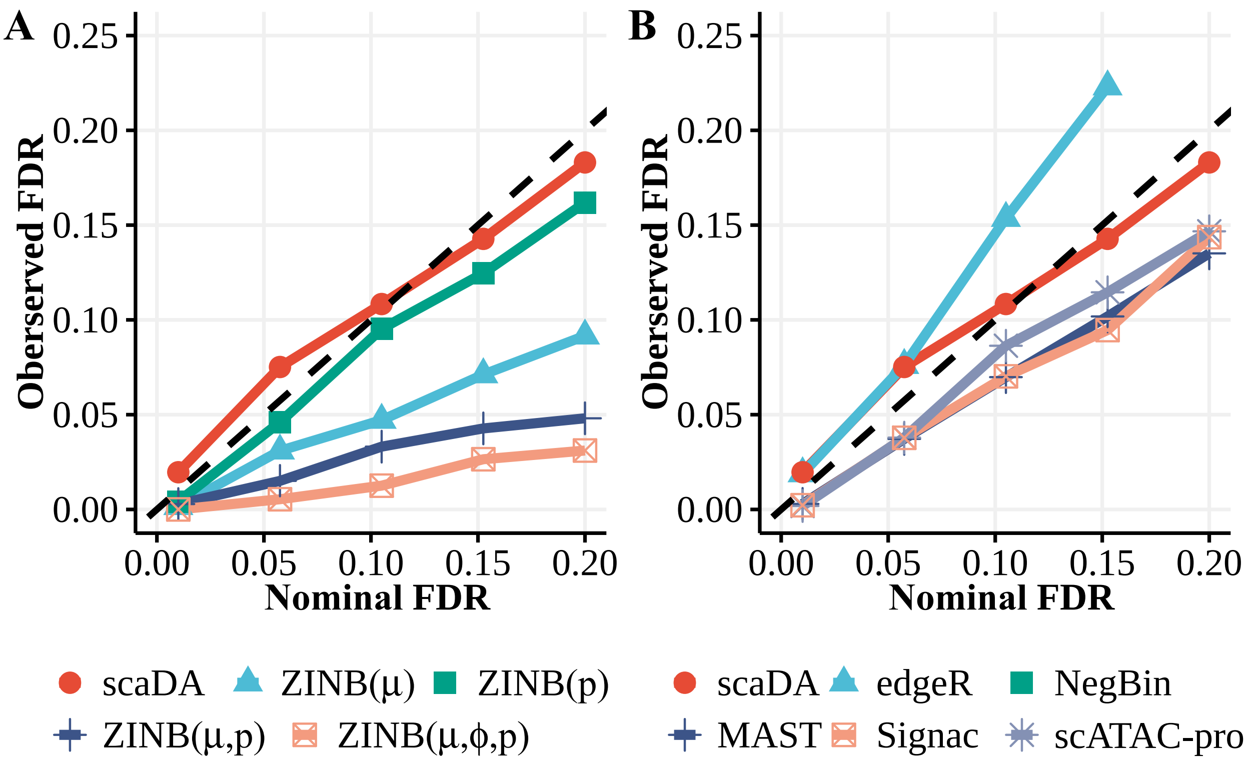

S6 Fig. FDR control analysis (Sample size 200 in each group). scaDA is compared to both ZINB-based LRTs and published methods. Using the same simulation strategy in Scenario 4 (log2FC=2.5), we assume 20% DA peaks and simulate the read counts based on ZINB for 4000 peaks across 200 cells in each group. The observed FDR is plotted against the nominal FDR level. **A.** scaDA is compared to ZINB-based LRT tests. **B.** scaDA is compared to published methods.
