# Supplementary material for: scaDA: A novel statistical method for differential analysis of single-cell chromatin accessibility sequencing data
Source: PLoS Comput Biol. 2024 Aug 2;20(8):e1011854. doi: 10.1371/journal.pcbi.1011854 (PMC11324137; doi:10.1371/journal.pcbi.1011854)
Supplement: S8 Fig — (PDF) [file pcbi.1011854.s009.pdf]

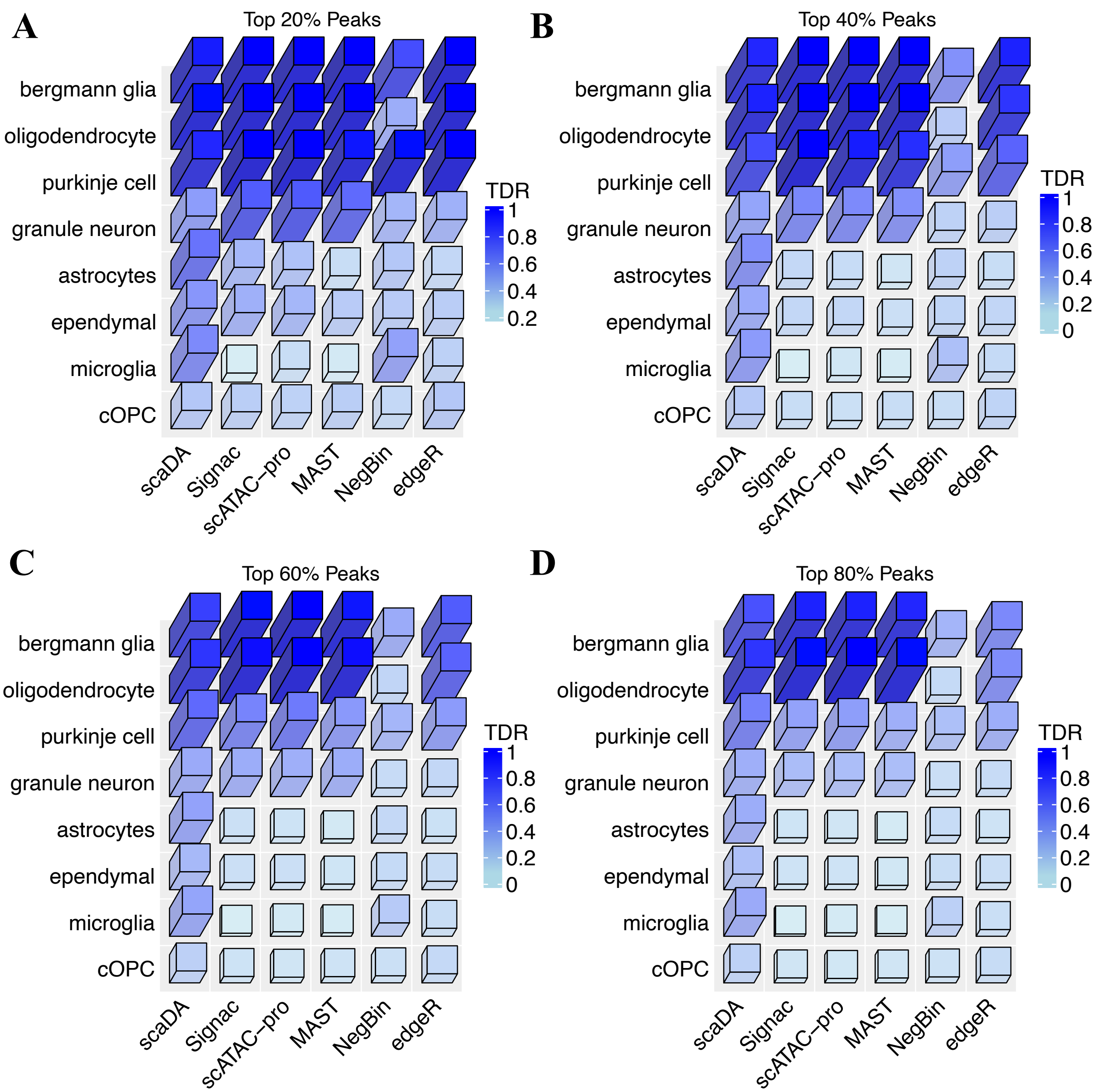

S8 Fig. Human Brain 3K: TDR across 8 cell types for scaDA and published methods at different levels of top percentages (20%, 40%, 60%, 80%)
