# Supplementary material for: scaDA: A novel statistical method for differential analysis of single-cell chromatin accessibility sequencing data
Source: PLoS Comput Biol. 2024 Aug 2;20(8):e1011854. doi: 10.1371/journal.pcbi.1011854 (PMC11324137; doi:10.1371/journal.pcbi.1011854)
Supplement: S1 Table — (PDF) [file pcbi.1011854.s015.pdf]

S1 Table. Human Brain 3K: Cell types, cell numbers and cell type proportion

celltype	cellnum	cell Proportion
granule neuron	636	22%
oligodendrocyte	552	20%
cOPC	365	13%
bergmann glia	344	12%
ependymal	204	7%
purkinje cell	144	5%
astrocytes	125	4%
microglia	104	4%
GCP	93	3%
brainstem	73	3%
MLI	69	2%
rhombic lip	63	2%
iCN	29	1%
UBC	29	1%
