# Supplementary material for: scaDA: A novel statistical method for differential analysis of single-cell chromatin accessibility sequencing data
Source: PLoS Comput Biol. 2024 Aug 2;20(8):e1011854. doi: 10.1371/journal.pcbi.1011854 (PMC11324137; doi:10.1371/journal.pcbi.1011854)
Supplement: S2 Table — (PDF) [file pcbi.1011854.s016.pdf]

S2 Table. Human Brain 3K: Mean of TDR across all cell types of scaDA and published methods at different levels of top percentages

Top Peaks	scaDA	Signac	scATAC-pro	MAST	NegBin	edgeR
20%	0.81	0.72	0.72	0.67	0.64	0.67
40%	0.73	0.58	0.58	0.56	0.42	0.54
60%	0.66	0.48	0.49	0.46	0.32	0.44
80%	0.59	0.42	0.42	0.40	0.26	0.35
100%	0.51	0.34	0.34	0.33	0.22	0.28
