# Supplementary material for: scaDA: A novel statistical method for differential analysis of single-cell chromatin accessibility sequencing data
Source: PLoS Comput Biol. 2024 Aug 2;20(8):e1011854. doi: 10.1371/journal.pcbi.1011854 (PMC11324137; doi:10.1371/journal.pcbi.1011854)
Supplement: S3 Table — (PDF) [file pcbi.1011854.s017.pdf]

**S3 Table. Human Brain 3K: Variance of TDR across all cell types of scaDA and published methods at different levels of top percentages**

| Top Peaks | scaDA | Signac | scATAC-pro | MAST | NegBin | edgeR |
|---|---|---|---|---|---|---|
| 20% | 0.03 | 0.09 | 0.08 | 0.10 | 0.05 | 0.08 |
| 40% | 0.05 | 0.15 | 0.15 | 0.16 | 0.03 | 0.11 |
| 60% | 0.05 | 0.15 | 0.15 | 0.15 | 0.02 | 0.09 |
| 80% | 0.05 | 0.14 | 0.14 | 0.14 | 0.01 | 0.06 |
| 100% | 0.03 | 0.09 | 0.09 | 0.10 | 0.01 | 0.03 |
