# Supplementary material for: scaDA: A novel statistical method for differential analysis of single-cell chromatin accessibility sequencing data
Source: PLoS Comput Biol. 2024 Aug 2;20(8):e1011854. doi: 10.1371/journal.pcbi.1011854 (PMC11324137; doi:10.1371/journal.pcbi.1011854)
Supplement: S4 Table — (PDF) [file pcbi.1011854.s018.pdf]

S4 Table. Human Brain 3K: Rank of scaDA and published methods by power

	scaDA	Signac	scATAC-pro	MAST	NegBin	edgeR
Rank 1	6	2	0	1	0	0
Rank 2	0	1	2	0	3	1
Rank 3	0	1	1	2	1	3
Rank 4	2	3	2	0	0	1
Rank 5	0	0	2	3	0	3
Rank 6	0	1	1	2	4	0
