# Supplementary material for: scaDA: A novel statistical method for differential analysis of single-cell chromatin accessibility sequencing data
Source: PLoS Comput Biol. 2024 Aug 2;20(8):e1011854. doi: 10.1371/journal.pcbi.1011854 (PMC11324137; doi:10.1371/journal.pcbi.1011854)
Supplement: S5 Table — (PDF) [file pcbi.1011854.s019.pdf]

S5 Table. Human PBMC 10K: Cell types, cell numbers and cell type proportion

celltype	cellnum	cellprop
CD4 TCM	2303	27%
CD14 Mono	2124	25%
CD4 Naive	858	10%
CD8 TEM	801	9%
CD8 Naive	515	6%
NK	336	4%
B intermediate	318	4%
CD16 Mono	266	3%
Treg	187	2%
CD4 TEM	154	2%
MAIT	144	2%
cDC2	119	1%
B naive	112	1%
gdT	111	1%
CD8 TCM	97	1%
B memory	85	1%
pDC	43	0%
CD4 CTL	24	0%
NK_CD56bright	21	0%
Plasmablast	16	0%
