# Supplementary material for: scaDA: A novel statistical method for differential analysis of single-cell chromatin accessibility sequencing data
Source: PLoS Comput Biol. 2024 Aug 2;20(8):e1011854. doi: 10.1371/journal.pcbi.1011854 (PMC11324137; doi:10.1371/journal.pcbi.1011854)
Supplement: S6 Table — (PDF) [file pcbi.1011854.s020.pdf]

S6 Table. Human PBMC 10K: Mean of TDR across all cell types for scaDA and published methods at different levels of top percentages

Top Peaks	scaDA	NegBin	edgeR	Signac	scATAC-pro	MAST
20%	0.57	0.49	0.45	0.45	0.43	0.40
40%	0.43	0.31	0.29	0.25	0.24	0.22
60%	0.34	0.24	0.21	0.17	0.16	0.15
80%	0.29	0.19	0.17	0.12	0.12	0.11
100%	0.24	0.16	0.14	0.10	0.10	0.09
