# Supplementary material for: scaDA: A novel statistical method for differential analysis of single-cell chromatin accessibility sequencing data
Source: PLoS Comput Biol. 2024 Aug 2;20(8):e1011854. doi: 10.1371/journal.pcbi.1011854 (PMC11324137; doi:10.1371/journal.pcbi.1011854)
Supplement: S7 Table — (PDF) [file pcbi.1011854.s021.pdf]

S7 Table. Human PBMC 3K: Variance of TDR across all cell types for scaDA and published methods at different levels of top percentages

Top Peaks	scaDA	NegBin	edgeR	Signac	scATAC-pro	MAST
20%	0.02	0.07	0.06	0.07	0.07	0.08
40%	0.02	0.04	0.03	0.03	0.03	0.03
60%	0.01	0.02	0.02	0.01	0.01	0.01
80%	0.01	0.02	0.01	0.01	0.01	0.01
100%	0.01	0.01	0.01	0.01	0.01	0.01
