# Supplementary material for: scaDA: A novel statistical method for differential analysis of single-cell chromatin accessibility sequencing data
Source: PLoS Comput Biol. 2024 Aug 2;20(8):e1011854. doi: 10.1371/journal.pcbi.1011854 (PMC11324137; doi:10.1371/journal.pcbi.1011854)
Supplement: S8 Table — (PDF) [file pcbi.1011854.s022.pdf]

S8 Table. Human Brain 3K: Rank of scaDA and published methods by power

	scaDA	NegBin	edgeR	Signac	scATAC-pro	MAST
Rank 1	9	5	0	0	0	0
Rank 2	5	8	1	1	0	0
Rank 3	0	0	12	1	0	0
Rank 4	0	1	0	10	6	4
Rank 5	0	0	1	2	6	0
Rank 6	0	0	0	0	2	10
