# Supplementary material for: scaDA: A novel statistical method for differential analysis of single-cell chromatin accessibility sequencing data
Source: PLoS Comput Biol. 2024 Aug 2;20(8):e1011854. doi: 10.1371/journal.pcbi.1011854 (PMC11324137; doi:10.1371/journal.pcbi.1011854)
Supplement: S9 Table — (PDF) [file pcbi.1011854.s023.pdf]

S9 Table. Human AD: experimental design

Batch	Control	Case
1	Sample-96	Sample-43
1	Sample-100	Sample-45
2	Sample-82	Sample-46
2	Sample-66	Sample-40
2	Sample-101	Sample-27
2		Sample-50
2		Sample-22
3	Sample-90	Sample-37
3	Sample-52	Sample-33
3	Sample-58	Sample-47
3		Sample-17
3		Sample-19
