# Supplementary material for: scaDA: A novel statistical method for differential analysis of single-cell chromatin accessibility sequencing data
Source: PLoS Comput Biol. 2024 Aug 2;20(8):e1011854. doi: 10.1371/journal.pcbi.1011854 (PMC11324137; doi:10.1371/journal.pcbi.1011854)
Supplement: S10 Table — (PDF) [file pcbi.1011854.s024.pdf]

S10 Table. Human AD: Mean of TDR across all cell types for scaDA and published methods at different levels of top percentages

Top peaks	scaDA	scATAC-pro	MAST	edgeR	Signac	NegBin
20%	0.77	0.65	0.62	0.51	0.45	0.16
40%	0.75	0.54	0.52	0.43	0.35	0.12
60%	0.73	0.46	0.44	0.39	0.30	0.10
80%	0.71	0.40	0.38	0.37	0.25	0.09
100%	0.69	0.34	0.33	0.35	0.21	0.09
