# Supplementary material for: scaDA: A novel statistical method for differential analysis of single-cell chromatin accessibility sequencing data
Source: PLoS Comput Biol. 2024 Aug 2;20(8):e1011854. doi: 10.1371/journal.pcbi.1011854 (PMC11324137; doi:10.1371/journal.pcbi.1011854)
Supplement: S11 Table — (PDF) [file pcbi.1011854.s025.pdf]

S11 Table. Human AD: Variance of TDR across all cell types for scaDA and published methods at different levels of top percentages

Top peaks	scaDA	scATAC-pro	MAST	edgeR	Signac	NegBin
20%	0.10	0.17	0.17	0.16	0.19	0.07
40%	0.10	0.17	0.17	0.14	0.17	0.05
60%	0.10	0.16	0.16	0.13	0.14	0.04
80%	0.10	0.14	0.14	0.12	0.12	0.04
100%	0.09	0.11	0.11	0.11	0.09	0.04
