# Supplementary material for: scaDA: A novel statistical method for differential analysis of single-cell chromatin accessibility sequencing data
Source: PLoS Comput Biol. 2024 Aug 2;20(8):e1011854. doi: 10.1371/journal.pcbi.1011854 (PMC11324137; doi:10.1371/journal.pcbi.1011854)
Supplement: S12 Table — (PDF) [file pcbi.1011854.s026.pdf]

S12 Table. Human AD: Rank of scaDA and published methods by mean power

	scaDA	scATAC-pro	MAST	edgeR	Signac	NegBin
Rank 1	6	0	0	0	0	0
Rank 2	0	3	1	2	0	0
Rank 3	0	3	2	1	0	0
Rank 4	0	0	3	3	0	0
Rank 5	0	0	0	0	6	0
Rank 6	0	0	0	0	0	6
